# Effect of peginterferon beta-1a on MRI measures and achieving no evidence of disease activity: results from a randomized controlled trial in relapsing-remitting multiple sclerosis

**DOI:** 10.1186/s12883-014-0240-x

**Published:** 2014-12-31

**Authors:** Douglas L Arnold, Peter A Calabresi, Bernd C Kieseier, Sarah I Sheikh, Aaron Deykin, Ying Zhu, Shifang Liu, Xiaojun You, Bjoern Sperling, Serena Hung

**Affiliations:** Montreal Neurological Institute, McGill University, 3801 University St., WB321, Montreal, QC H3A 2B4 Canada; NeuroRx Research, Montreal, QC Canada; Department of Neurology, Johns Hopkins University, 600 N. Wolfe St., Pathology 627A, Baltimore, MD 21287 USA; Department of Neurology, Heinrich-Heine University, Moorenstrasse 5, Düsseldorf, 40225 Germany; Biogen Idec Inc., 14 Cambridge Center, Cambridge, MA 02142 USA

**Keywords:** Clinical trial, Multiple sclerosis, Pegylation, Interferon

## Abstract

**Background:**

Subcutaneous peginterferon beta-1a provided clinical benefits at Year 1 (placebo-controlled period) of the 2-Year Phase 3 ADVANCE study in relapsing-remitting multiple sclerosis (RRMS). Here we report the effect of peginterferon beta-1a on brain magnetic resonance imaging (MRI) lesions, and no evidence of disease activity (NEDA; absence of clinical [relapses and 12-week confirmed disability progression] and MRI [gadolinium-enhancing, and new or newly-enlarging T2 hyperintense lesions] disease activity) during Year 1.

**Methods:**

RRMS patients (18–65 years; Expanded Disability Status Scale score ≤5) were randomized to double-blind placebo or peginterferon beta-1a 125 μg every 2 or 4 weeks. Sensitivity analyses of last observation carried forward and composite disease activity (using minimal MRI allowance definitions) were conducted.

**Results:**

1512 patients were randomized and dosed (placebo n = 500; peginterferon beta-1a every 2 [n = 512] or 4 [n = 500] weeks). Every 2 week dosing significantly reduced, versus placebo and every 4 week dosing, the number of new or newly-enlarging T2 hyperintense lesions at Weeks 24 (by 61% and 51%, respectively) and 48 (secondary endpoint; by 67% and 54%, respectively); all p < 0.0001. Every 2 week dosing also provided significant reductions versus placebo and every 4 week dosing in the number of new T1 hypointense, gadolinium-enhancing, and new active (gadolinium-enhancing plus non-enhancing new T2) lesions (all p < 0.0001), as well as the volume of T2 and T1 lesions (p < 0.05) at Weeks 24 and 48. Significantly more patients dosed every 2 weeks had NEDA versus placebo and every 4 weeks (all p < 0.01) from baseline to Week 48 (33.9% versus 15.1% and 21.5%, respectively [odds ratios, ORs: 2.89 and 1.87]), from baseline to Week 24 (41.0% versus 21.9% and 30.7%, [ORs: 2.47 and 1.57]) and from Week 24 to Week 48 (60.2% versus 28.9% and 36.6%, [ORs: 3.71 and 2.62]). Consistent results were seen when allowing for minimal MRI activity.

**Conclusion:**

During Year 1 of ADVANCE, significantly more RRMS patients receiving peginterferon beta-1a every 2 weeks had NEDA, and early and sustained improvements in all MRI endpoints, versus placebo and every 4 week dosing. NEDA sensitivity analyses align with switch strategies in clinical practice settings and provide insight into future responders/non-responders.

**Trial registration:**

ClinicalTrials.gov: NCT00906399

**Electronic supplementary material:**

The online version of this article (doi:10.1186/s12883-014-0240-x) contains supplementary material, which is available to authorized users.

## Background

Subcutaneous (SC) peginterferon beta-1a, a pegylated form of interferon (IFN) beta-1a, is a new treatment for relapsing multiple sclerosis (MS) [1-3]. Pegylation (modification via attachment of polyethylene glycol [PEG] molecules), has been used to increase half-life and reduce the immunogenicity of certain drugs with either increased or sustained efficacy [4-9]. Results from the first year (placebo-controlled period prior to cross-over onto active drug) of the 2-year pivotal Phase 3, multicenter, randomized ADVANCE study in patients with relapsing-remitting MS (RRMS) showed that peginterferon beta-1a (125 μg SC) administered every 2 or 4 weeks significantly improved clinical endpoints, with a reduction in annualized relapse rate (primary endpoint), and risk of relapse and 12-week confirmed disability progression (both secondary endpoints), and several MRI-related endpoints versus placebo, with a safety profile reflecting established IFN beta-1a therapies [10]. Furthermore, every 2 week dosing provided numerically greater reductions relative to placebo versus every 4 week dosing across relapse and all MRI endpoints [10].

Magnetic resonance imaging (MRI) of lesions in the brain is an important tool for monitoring MS disease activity and progression [11,12], providing a sensitive measure of the effects of treatment on focal inflammatory activity in the central nervous system [13], which initiates the demyelination and axonal damage associated with MS disease progression [14,15]. Lesions detected on MRI scans have been shown to correlate with MS prognosis [16-18]; thus early reduction in MRI lesions is an important treatment goal [12].

With the development of effective biological therapies, no evidence of disease activity (NEDA; absence of clinical [defined as no relapses and no onset of 12-week confirmed disability progression] and MRI [no gadolinium-enhancing lesions and no new or newly-enlarging T2 hyperintense lesions] disease activity) is now achievable in many patients with RRMS [19]. As treatments have improved, annualized relapse rates recorded in clinical studies have decreased to the point where there is a need to consider alternative endpoints to evaluate new treatments for RRMS [20]. NEDA has been proposed for use as an endpoint in MS studies [20]. It has been evaluated in several MS studies to date [13,19,21,22], with some variation in its definition to allow for minimal MRI activity [23]. Although complete NEDA would be the ideal treatment aspiration, for many physicians, local guidelines and/or switch recommendations apply (e.g. two or more new or newly-enlarging T2-lesions or even a clinical relapse with incomplete remission before escalation is recommended) [24].

The objectives of the present analyses were to explore the effect of peginterferon beta-1a every 2 and 4 weeks on MS disease activity as measured by brain MRI lesions during Year 1 of the ADVANCE study versus placebo (at Week 48 ([Year 1], as previously reported [10], and at the earlier time point of Week 24), with presentation of new post-hoc statistical analyses for every 2 week versus every 4 week dose regimen comparisons at these time points, and to determine the effect of peginterferon beta-1a on the combined endpoint of NEDA via post-hoc investigations of Year 1 data (looking at a range of time-scales to evaluate timing of NEDA impact: baseline to Week 48, baseline to Week 24, and Week 24 to Week 48). Sensitivity analyses of disease activity were also carried out to include an allowance for patients who did not undergo all MRI measures during the study, but had no evidence of disease activity on any recorded assessment (last observation carried forward [LOCF] approach), and using alternative definitions that mimic thresholds used in clinical practice to guide decision-making on treatment continuation due to efficacy, or treatment switch due to inadequate efficacy (no clinical activity with minimal MRI activity).

## Methods

### Study design and participants

We conducted a randomized, multicenter (at 183 sites in 26 countries), double-blind, placebo-controlled, parallel group Phase 3 study of peginterferon beta-1a in patients with RRMS [10]. Key eligibility criteria were diagnosis of relapsing MS as defined by the McDonald criteria [25], an age of 18–65 years, a score of 0–5 on the Expanded Disability Status Scale (EDSS [26]), and at least two clinically documented relapses in the previous three years, with at least one of these relapses having occurred within 12 months prior to randomization. Key exclusion criteria were progressive forms of MS, pre-specified laboratory abnormalities, and prior interferon beta treatment for MS exceeding four weeks or discontinuation less than six months prior to baseline. The protocol was approved by each site’s institutional review board or ethics committee (a list of ethics committees for participating sites is provided in Additional file 1). The ethics committee at Johns Hopkins University (the site of the Principal Investigator) was Johns Hopkins Medicine Institutional Review Board, Baltimore, MD, USA. The study was conducted according to the International Conference on Harmonization Guidelines for Good Clinical Practice and the Declaration of Helsinki. Every patient provided written informed consent prior to study entry.

### Study procedures and endpoints

The methods of the ADVANCE study have been published previously [10]. Here, we focus on information specific to the analyses presented.

Patients were randomly assigned in a 1:1:1 ratio to receive SC injections of placebo (Year 1 only), peginterferon beta-1a at a dose of 125 μg every 2 weeks, or peginterferon beta-1a 125 μg every 4 weeks. Patients received either peginterferon beta-1a or placebo every 2 weeks to maintain the blind; those randomized to the peginterferon beta-1a every 4 week group received alternate injections of placebo and peginterferon beta-1a every 4 weeks. Participants randomized to receive peginterferon beta-1a at beginning of study, and those re-randomized to active drug after first year, underwent dose escalation over the first four weeks of the treatment to mitigate influenza-like symptoms (starting dose 63 μg; 94 μg at Week 2; target dose 125 μg at Week 4 and thereafter) [10]. The current analysis evaluates the effects of receiving peginterferon beta-1a vs. placebo in Year 1 (baseline to 48 weeks) of the ADVANCE study. All study management and site personnel, investigators, and patients were blinded to treatment assignment including dose escalation. Each site used separate examining and treating neurologists, thereby maintaining rater blinding for all treatment groups.

MRI scans were obtained at screening, and at Weeks 24, and 48, and were evaluated in a blinded manner at a central MRI reading center (NeuroRx Research, Montreal, Canada).

The number of new or newly-enlarging T2 hyperintense lesions at Week 48 compared with baseline was a secondary endpoint of the study. Tertiary MRI endpoints included the number of new or newly-enlarging T2 hyperintense lesions at Week 24, the number of gadolinium-enhancing (Gd+), new T1 hypointense, and new active (sum of Gd+ plus non-enhancing new or newly-enlarging T2 hyperintense) lesions, at Weeks 24 and 48, and the volume of T2 and T1 hypointense lesions, at Weeks 24 and 48.

Post-hoc NEDA analyses during Year 1 of the ADVANCE study were carried out and summarized as data from baseline to Week 48, baseline to Week 24 and Week 24 to Week 48 using the following definitions: NEDA was defined as absence of both clinical [no relapses and no onset of 12-week confirmed disability progression over the interval] and MRI [no Gd+ lesions and no new or newly-enlarging T2 hyperintense lesions] disease activity during the respective time periods; MRI-NEDA only was defined as no Gd+ lesions at Weeks 24 and 48 and no new or newly-enlarging T2 lesions at Week 48 compared with baseline (for data summarized from baseline to Week 48), no Gd+ lesions at Week 24 and no new or newly-enlarging T2 lesions at Week 24 compared with baseline (for data summarized from baseline to Week 24), and no Gd+ at Week 48 and no new or newly-enlarging T2 lesions at Week 48 compared with Week 24 or the closest previous visit before Week 48 (for data summarized from Week 24 to Week 48); clinical-NEDA only was defined as no relapses and no onset of 12-week confirmed disability progression over the intervals specified (including 12 weeks confirmation at Week 60). Sensitivity analyses were also carried out on data from baseline to Week 48 to include an allowance for patients with missing measures of MRI disease activity (analysis 1 below) and using definitions with an allowance for minimal MRI activity (analyses 2 and 3 below):

- Sensitivity analysis 1: definitions described above were applied using LOCF (i.e. patients were not excluded from overall NEDA analyses if they did not have complete MRI scan data during Year 1, and were not excluded from MRI-NEDA only analyses if they only had partial MRI scan data during Year 1; patients were excluded from MRI-NEDA only analyses if they were missing all MRI scan data during Year 1).
- Sensitivity analysis 2: sum of number of new or newly-enlarging T2 lesions at Week 48 compared with baseline and Gd+ lesions at both Week 24 and Week 48 ≤2.
- Sensitivity analysis 3: ≤1 new or newly-enlarging T2 lesion at Week 48 compared with baseline and no Gd+ lesions at Weeks 24 and 48.

### Statistical analysis

Statistical tests were two-sided. Multiple comparisons adjustments were performed on the primary and secondary endpoints for the study, with an overall type I error rate of 0.05. All primary and secondary endpoints for the study were statistically significant for both dose frequencies [10]. However no multiple comparisons adjustments were performed on tertiary endpoints or post-hoc analyses, so nominal p-values are reported here for each endpoint.

MRI results were derived from patients who had any MRI data (a majority of the overall study intention-to-treat [ITT] population [randomized patients who received at least one dose of study treatment]) [10]. Negative binomial regression was used for analysis of new or newly-enlarging hyperintense lesions on T2-weighted images (adjusted for baseline number of T2 lesions) and new active lesions (adjusted for baseline number of Gd+ lesions); multiple logit regression was used for the analysis of Gd+ and new T1-hypointense lesions (adjusted for baseline number of respective lesions) [10]. Analysis of covariance (ANCOVA) was used for analysis of lesion volumes, adjusted for respective baseline lesion volumes [10]. Comparisons of active treatment dosing regimens versus placebo were pre-specified; every 2 week versus every 4 week comparisons were post-hoc. Post-hoc NEDA and disease activity sensitivity analyses (no clinical activity with minimal MRI activity) proportions were calculated directly based on the definitions using data from all eligible patients. A logistic regression model was used to calculate odds ratios (ORs) and corresponding p-values.

## Results

### Patient disposition and baseline characteristics

A total of 1516 patients underwent randomization, of whom 1512 received at least one dose of study drug and were included in the ITT population (placebo n = 500; peginterferon beta-1a every 2 weeks n = 512; peginterferon beta-1a every 4 weeks n = 500) [10]. Patient demographics and baseline disease characteristics in the overall study population were generally well balanced across the treatment groups [10]; data are summarized in Table 1. Patients included in ADVANCE had mean baseline EDSS scores of 2.44−2.48; 17% had been treated with any MS medication prior to study entry, including 7% who used beta interferons or glatiramer acetate. Compared with the placebo and peginterferon beta-1a every 4 week groups, slightly more subjects in the peginterferon beta-1a every 2 week group had no Gd+ lesions at baseline (59%, 59%, and 65%, respectively; Table 1) and mean number of Gd+ lesions at baseline was also lower in this treatment group (Table 1); as mentioned above, analyses of Gd+ lesion number were adjusted for baseline Gd+ number of lesions. The overall study completion rate for Year 1 was 88% (a complete overview of ADVANCE Year 1 patient disposition has been published previously [10]).

**Table 1:** **Patient demographics and baseline disease characteristics of the ITT population** [10]

**Characteristic**	**Placebo (n = 500)**	**Peginterferon beta-1a 125 μg every 4 weeks**	**Peginterferon beta-1a 125 μg every 2 weeks**
		**(n = 500)**	**(n = 512)**
Age, years	36.3 (9.74)	36.4 (9.87)	36.9 (9.79)
Gender, % female	72	70	71
Time since first MS symptoms, years	6.3 (6.28)	6.5 (6.07)	6.9 (6.61)
Relapses within the previous 12 months	1.6 (0.67)	1.5 (0.62)	1.6 (0.67)
EDSS Score	2.44 (1.18)	2.48 (1.24)	2.47 (1.26)
Number who took any prior MS medication^a^, n (%)	86 (17)	85 (17)	89 (17)
No Gd+ lesions, n (%)	296 (59)	297 (59)	334 (65)
Number of T2 lesions	50.6 (35.7)	51.4 (36.0)	48.7 (36.8)
Number of T1 hypointense lesions	28.1 (29.2)	29.6 (30.8)	27.8 (28.1)
Number of Gd+ lesions	1.6 (3.81)	1.8 (5.38)	1.2 (3.44)
Volume of T2 lesions, cm^3^	10.1 (11.9)	11.3 (13.2)	9.8 (11.6)
Volume of T1 hypointense lesions, cm^3^	3.1 (4.80)	3.1 (4.76)	3.0 (4.50)

MRI results are reported for a slightly smaller number of patients compared with the ITT population (due to the availability of MRI scans to analyze). Data are presented as mean (standard deviation), unless otherwise stated. ^a^Interferon beta-1a, interferon beta-1b, or glatiramer acetate.

EDSS = Expanded Disability Status Scale; Gd+ = gadolinium-enhancing; MS = multiple sclerosis.

### MRI outcomes

**Figure 1:**
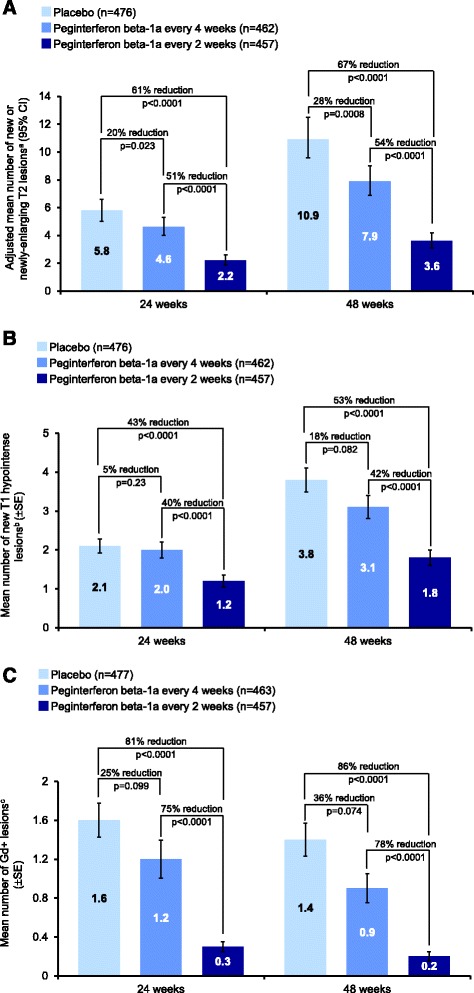
**Lesion numbers at Weeks 24 and 48. A)** New or newly-enlarging T2 lesions ^a^Based on negative binomial regression analysis, adjusted baseline T2 lesion number. The data for 48 weeks has been published previously [10]. CI = confidence interval. **B)** New T1 hypointense lesions compared to baseline ^b^Based on mean number of new lesions only and p value based on multiple logit regression, adjusted for baseline number of T1 lesions. The data for 48 weeks has been published previously [10]. SE = standard error. **C)** Gd+ lesions ^c^Based on mean number of lesions only and p value based on multiple logit regression, adjusted for baseline number of Gd+ lesions. Gd+ = gadolinium-enhancing; SE = standard error.

Peginterferon beta-1a every 2 weeks significantly reduced the number of new or newly-enlarging T2 lesions, new T1 hypointense lesions, Gd+ lesions, and new active lesions at Weeks 24 and 48, compared with placebo and peginterferon beta-1a every 4 weeks (all p < 0.0001; Figure 1). Peginterferon beta-1a every 4 weeks significantly reduced the number of new or newly-enlarging T2 lesions (Figure 1) and new active lesions at Weeks 24 and 48, compared with placebo (Additional file 2), and numerically reduced the number of new T1 hypointense lesions and Gd+ lesions, although reductions did not reach statistical significance (Figure 1).

Peginterferon beta-1a every 2 weeks significantly reduced the volume of T2 and T1 hypointense lesions at Weeks 24 and 48, compared with placebo and peginterferon beta-1a every 4 weeks (Table 2). Peginterferon beta-1a every 4 weeks significantly reduced the volume of T2 lesions at Weeks 24 and 48, compared with placebo (Table 2); no significant reduction in the volume of T1 hypointense lesions was seen versus placebo at Weeks 24 or 48 for every 4 week dosing (Table 2).

**Table 2:** **Lesion volumes at Weeks 24 and 48 compared to baseline**

**Characteristic**	**Placebo (n = 476)**	**Peginterferon beta-1a 125 μg every 4 weeks**	**Peginterferon beta-1a 125 μg every 2 weeks**
		**(n = 462)**	**(n = 457)**
Mean change from baseline in T2 lesion volume, cm^3^, at 24 weeks	0.34	0.14	−0.22
P value vs. placebo		p = 0.0006	p < 0.0001
P value vs. every 4 weeks	-	-	p < 0.0001
Mean change from baseline in T2 lesion volume, cm^3^, at 48 weeks	0.77	0.06	−0.26
P value vs. placebo		p < 0.0001	p < 0.0001
P value vs. every 4 weeks	-	-	p < 0.0001
Mean change from baseline in TI hypointense lesion volume, cm^3^, at 24 weeks	0.29	0.31	0.18
P value vs. placebo		p = 0.22	p = 0.0002
P value vs. every 4 weeks	-	-	p = 0.014
Mean change from baseline in TI hypointense lesion volume, cm^3^, at 48 weeks	0.54	0.57	0.32
P value vs. placebo		p = 0.18	p < 0.0001
P value vs. every 4 weeks	-	-	p = 0.0032

### Analyses of achieving no evidence of disease activity

**Figure 2:**
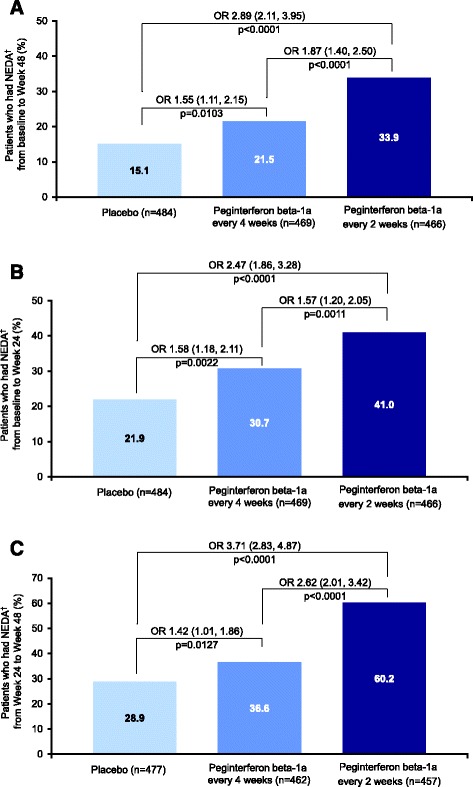
**NEDA proportions. A)** Baseline to Week 48. **B)**. Baseline to Week 24. **C)**. Weeks 24–48. ORs are shown with 95% confidence intervals in parentheses. ^a^Defined as absence of both clinical (no relapses and no onset of 12-week confirmed disability progression over the interval) and MRI (no gadolinium-enhancing lesions and no new or newly-enlarging T2 hyperintense lesions) disease activity; data from patients with complete MRI results during the time interval were used for analysis of MRI disease activity. NEDA = No evidence of disease activity; MRI = magnetic resonance imaging; OR = odds ratio.

Significantly higher proportions of patients receiving peginterferon beta-1a every 2 and every 4 weeks had NEDA during each assessment period, compared with placebo (baseline to Week 48 ORs: 2.89 [p < 0.0001] and 1.55 [p = 0.0103], respectively; baseline to Week 24 ORs: 2.47 [p < 0.0001] and 1.58 [p = 0.0022], respectively; Weeks 24–48 ORs: 3.71 [p < 0.001] and 1.42 [p = 0.0127], respectively; Figure 2). Significantly more patients had NEDA on every 2 week versus every 4 week dosing (baseline to Week 48: OR 1.87 [p < 0.0001]; baseline to Week 24: OR 1.57 [p = 0.0011]; Weeks 24–48: OR 2.62 [p < 0.001]; Figure 2). Less measured disease activity was observed in Weeks 24–48 than in the first 24 weeks for the every 2 week dosing group.

Significantly higher proportions of patients receiving peginterferon beta-1a every 2 and every 4 weeks had MRI-NEDA during each assessment period, compared with placebo (baseline to Week 48 ORs: 2.94 [p < 0.0001] and 1.41 [p = 0.0318], respectively; baseline to Week 24 ORs: 2.44 [p < 0.0001] and 1.46 [p = 0.0078], respectively; Weeks 24–48 ORs: 4.11 [p < 0.0001] and 1.44 [p = 0.0080], respectively; Additional file 2). Significantly more patients had MRI-NEDA on every 2 week versus every 4 week dosing (baseline to Week 48: OR 2.09 [p < 0.0001]; baseline to Week 24: OR 1.67 [p = 0.0002]; Weeks 24–48 OR: 2.86 [p < 0.0001]; Additional file 2).

Significantly greater proportions of patients receiving peginterferon beta-1a every 2 and every 4 weeks had clinical-NEDA from baseline to Week 48 and from baseline to Week 24, compared with placebo (baseline to Week 48 ORs: 1.77 [p < 0.0001] and 1.48 [p = 0.0060], respectively; baseline to Week 24 ORs: 1.57 [p = 0.0088] and 1.61 [p = 0.0063], respectively; Additional file 2). From Weeks 24 to 48, significantly greater proportions of patients receiving peginterferon beta-1a every 2 weeks had clinical-NEDA, compared with placebo (OR: 1.75 [p = 0.0070]; every 4 weeks vs. placebo OR: 1.22 [p = 0.3058]; Additional file 2). No significant difference was seen between every 2 week and every 4 week dosing for proportions of clinical-NEDA patients (baseline to Week 48: OR 1.20 [p = 0.2367]; baseline to Week 24: OR 0.98 [p = 0.8947]; Weeks 24–48 OR: 1.44 [p = 0.0898]; Additional file 2).

Results from disease activity sensitivity analyses involving an allowance for missing MRI scan data, or defined as no clinical activity with allowances for minimal MRI activity, were consistent with the primary NEDA analyses, with higher ORs for peginterferon beta-1a every 2 weeks, favoring this dosing regimen over every 4 weeks and placebo (Table 3).

**Table 3:** **Sensitivity analyses of disease activity at Week 48**

	**Placebo**	**Peginterferon beta-1a every 4 weeks**	**Peginterferon beta-1a every 2 weeks**
**Sensitivity analysis 1** ^**a**^			
NEDA			
n/N (%)	89/500 (17.8)	132/500 (26.4)	204/512 (39.8)
OR (95% CI) vs. placebo	-	1.66 (1.22, 2.24)	3.06 (2.29, 4.09)
		p = 0.0011	p < 0.0001
OR (95% CI) vs. every 4 weeks	-	-	1.85 (1.42, 2.41)
			p < 0.0001
MRI-NEDA only			
n/N (%)	91/477 (19.1)	116/463 (25.1)	187/457 (40.9)
OR (95% CI) vs. placebo	-	1.42 (1.04, 1.93)	2.94 (2.19, 3.95)
		p = 0.0274	p < 0.0001
OR (95% CI) vs. every 4 weeks	-	-	2.07 (1.56, 2.74)
			p < 0.0001
**Sensitivity analysis 2** ^**b**^			
No clinical activity + minimal MRI activity			
n/N (%)	129/483 (26.7)	160/469 (34.1)	251/466 (53.9)
OR (95% CI) vs. placebo	-	1.42 (1.08, 1.88)	3.20 (2.44, 4.20)
		p = 0.0131	p < 0.0001
OR (95% CI) vs. every 4 weeks	-	-	2.25 (1.73, 2.94)
			p < 0.0001
Minimal MRI activity			
n/N (%)	161/476 (33.8)	194/462 (42.0)	295/457 (64.6)
OR (95% CI) vs. placebo	-	1.42 (1.09, 1.85)	3.56 (2.72, 4.67)
		p = 0.0100	p < 0.0001
OR (95% CI) vs. every 4 weeks	-	-	2.52 (1.93, 3.28)
			p < 0.0001
**Sensitivity analysis 3** ^**c**^			
No clinical activity + minimal MRI activity			
n/N (%)	104/484 (21.5)	135/469 (28.8)	214/466 (45.9)
OR (95% CI) vs. placebo	-	1.48 (1.10, 1.98)	3.10 (2.34, 4.12)
		p = 0.0096	p < 0.0001
OR (95% CI) vs. every 4 weeks	-	-	2.10 (1.60, 2.75)
			p < 0.0001
Minimal MRI activity			
n/N (%)	129/477 (27.0)	158/462 (34.2)	251/457 (54.9)
OR (95% CI) vs. placebo	-	1.40 (1.06, 1.85)	3.29 (2.50, 4.32)
		p = 0.0175	p < 0.0001
OR (95% CI) vs. every 4 weeks	-	-	2.34 (1.80, 3.06)
			p < 0.0001

^a^LOCF (i.e. patients were not excluded from NEDA analyses if they had missing MRI scan data during Year 1, and were not excluded from MRI-NEDA only analyses if they had data from ≥1 MRI scan during Year 1); ^b^Defined as the total number of new or newly-enlarging T2 lesions at Week 48 compared to baseline and the number of gadolinium-enhancing lesions at both Week 24 and Week 48 ≤2; ^**c**^Defined as ≤1 new or newly-enlarging T2 lesion at Week 48 compared with baseline and no gadolinium-enhancing lesions at Weeks 24 and 48. CI = confidence interval; LOCF = last observation carried forward; n/N = patients free from disease activity/total number of patients analyzed; OR = odds ratio.

## Discussion

The MRI outcomes of peginterferon beta-1a observed here are consistent with the robust benefits on clinical outcomes after one year of treatment (reductions in risk of 12-week confirmed disability progression [every 2 and 4 weeks 38%], risk of relapse [every 2 weeks 39%; every 4 weeks 26%], and annualized relapse rate [every 2 weeks 36%; every 4 weeks 28%]), as reported previously for the Phase 3 ADVANCE study [10]. Peginterferon beta-1a every 2 weeks provided superior outcomes for MRI lesion-based endpoints (including numbers of new or newly-enlarging T2 lesions, new T1 lesions, Gd+ lesions, and new active lesions, and volumes of new or newly-enlarging T2 lesions, and new T1 lesions) versus placebo and versus peginterferon beta-1a every 4 weeks, with statistical significance observed at the first brain MRI scheduled at Week 24 of treatment, and greater reductions through to Week 48 of the ADVANCE study. Peginterferon beta-1a every 4 weeks also provided superior results versus placebo at Weeks 24 and 48 for numbers of new or newly-enlarging T2 lesions and new active lesions, and the volume of T2 lesions, but not for numbers of Gd+ or T1 lesions, nor volume of T1 lesions.

An early decrease in brain inflammation, as shown by reductions in MRI disease activity, is particularly desirable in MS, as it may impact on associated injury to myelin and axons, with consequences for the accumulation of disability [12]. Indeed, MRI lesion measures have been shown to be strong predictors of clinical prognosis for MS patients [15-18,27,28].

This study also carried out post-hoc analyses of ADVANCE Year 1 data using composites of clinical and radiological indicators of disease activity, which are commonly used as endpoints in MS clinical trials [29-33]. Efficacy data show that proportions of patients with NEDA and MRI-NEDA only) were significantly higher with peginterferon beta-1a every 2 weeks from baseline to Week 48, from baseline to Week 24 and from Weeks 24 to 48, versus placebo and peginterferon beta-1a every 4 weeks. Furthermore, the sensitivity analyses of disease activity conducted support the robustness of the results of the primary NEDA analyses, indicating that the effects of peginterferon beta-1a are sustained when including an allowance for patients who did not undergo all MRI measures during the study, but had no evidence of disease activity on any recorded assessment, and when applying definitions with allowances for minimal MRI activity. These observations are important because brain MRIs within 12 months of starting a disease-modifying therapy are a sensitive tool in predicting response to treatment, along with the early identification of patients who will develop long-term disability progression [15,27,28,34]. MRI allowance is pertinent to current practice, as scans obtained 6–12 months after starting a new therapy may include MRI activity occurring before efficacy of a new therapy is apparent. Of note, fewer lesions were seen in the present study from Weeks 24–48, versus baseline to Week 24, and comparative ORs for NEDA and MRI-NEDA were higher from Week 24 to 48, compared with baseline to Week 24 and baseline to Week 48. Therefore, an MRI baseline established 6 months after treatment initiation may more effectively demonstrate differences between treatment arms. Further, activity on MRI may also indicate a need to switch to a treatment with an alternative mode of action, with the aim of avoiding irreversible clinical neurological disability [34,35]; however this need may be more appropriately assessed by establishing a baseline for new disease activity (e.g. at 24 weeks) thus avoiding switch to potentially less safe treatments.

NEDA analyses in the ADVANCE study reflect efficacy levels previously reported in other Phase 3 trials of agents approved for the treatment of relapsing forms of MS, however, it is important to consider that direct comparisons across studies may not be advisable due to differences between study design, patient populations, MRI techniques and MRI reading techniques used across different studies. In the FREEDOMS II study in RRMS, at Month 24 significantly more patients had freedom from indicators of new inflammatory activity (i.e. no gadolinium-enhanced T1 lesions and no new or newly-enlarged T2 lesions) on fingolimod (0.5 mg 50%; 1.25 mg 63%) compared with those on placebo (26%); no composites of clinical-NEDA or complete NEDA were reported in the FREEDOMS II study [36]; although no OR were reported, the relative differences between placebo and actives arms are similar to the findings in the present study.

It is important to compare methods of analysis (e.g. LOCF or non-LOCF), sensitivity of MRI analyses (e.g. computerized or visual read of scans) and numbers of scans when trying to estimate efficacy by endpoints like NEDA, or FMDA (Freedom from Measured Disease Activity), as termed by others, among different treatment options; it would therefore be optimal to make comparisons versus placebo-treated patients and to also compare OR results, rather than just absolute numbers.

Further analyses will be necessary to determine whether these early patients who had NEDA in the ADVANCE study remain NEDA over the longer term (i.e., after 96 weeks). A multicenter, dose-frequency blinded minimum 2-year extension of the ADVANCE study is also underway (ATTAIN; NCT01332019), which may allow for the provision of 4-year data.

## Conclusion

Analyses of MRI outcomes and NEDA, along with efficacy for single clinical endpoints reported previously [10], support SC peginterferon beta-1a every 2 weeks as an effective treatment option for patients with RRMS, with the benefit of less frequent administration.

## Additional files

Additional file 1:
**List of Ethics Committees, Study 105MS301.**
Additional file 2: Figure S1. Lesion numbers at Weeks 24 and 48. **Figure S2.** MRI-NEDA proportions. **Figure S3.** Clinical-NEDA proportions.

## Abbreviations

ANCOVA: Analysis of covariance
EDSS: Expanded Disability Status Scale
FMDA: Freedom from measured disease activity
Gd+: Gadolinium-enhancing
IFN: Interferon
ITT: Intention-to-treat
LOCF: Last observation carried forward
MRI: Magnetic resonance imaging
MS: Multiple sclerosis
NEDA: No evidence of disease activity
OR: Odds ratio
PEG: Polyethyleneglycol
RRMS: Relapsing-remitting MS
SC: Subcutaneous
