# Supplementary material for: Effect of peginterferon beta-1a on MRI measures and achieving no evidence of disease activity: results from a randomized controlled trial in relapsing-remitting multiple sclerosis
Source: BMC Neurol. 2014 Dec 31;14:240. doi: 10.1186/s12883-014-0240-x (PMC4311432; doi:10.1186/s12883-014-0240-x)
Supplement: Additional file 1: — List of Ethics Committees, Study 105MS301. [file 12883_2014_240_MOESM1_ESM.docx]

**Additional File 1:** List of Ethics Committees, Study 105MS301

| **Belgium** |
| --- |
| - Comité d'Ethique UCL Saint-Luc, Avenue Hippocrate 55.14, Tour Havey, niveau 0, Brussels 1200, Belgium |
| - Regionaal Ziekenhuis Sint-Trudo VZW - Ethisch comité, Diestersteenweg 100, Campus Sint-Jozef, Sint-Truiden, 3800, Belgium |
|  |
| **Bulgaria** |
| - Ethics Committee for Multi-centre Trials, 8 Damyan Gruev Str., Sofia, 1303, Bulgaria |
|  |
| **Canada** |
| - University of Western Ontario Health Sciences Research Ethics Board, 1393 Western Road, Room 4180, London, Ontario, N6G 1G9, Canada |
| - Comite d'Ethique-Hopital Saint Luc-Edifice Cooper, 3981 Boulevard St. Laurent, Mezzanine 2-Bureau M-207, Montreal, Quebec, Canada |
|  |
| **Chile** |
| - Comite de Evaluacion Etico Cientifico del Servicio de Salud Metropolitano Sur Oriente, Avenida Concha y Toro 3459, Oficina de Investigaciones Medicas, Block Central, Puente Alto, Santiago 8207257, Chile |
|  |
| **Colombia** |
| - EC of Fundacion del Caribe para la Investigacion Biomedica (Fundacion BIOS, Cra 44 No 72 -131 Oficina 201, Barranquilla, Colombia |
| - Comité de Ética e Investigaciones de la Fundación Clínica Abood Shaio, Diagonal 115A Numero 70C-75, Bogota, Colombia |
|  |
| **Croatia** |
| - Central Ethics Committee, Ksaver 200A, Zagreb, 10000, Croatia |
| - Local Drug Committee at Clinical Hospital "Sestre Milosrdnice", 29 Vinogradska Street, Zagreb, 10000, Croatia |
| - Local Drug Committee at Clinical Hospital "Dubrava", 6 Gojka Suska Avenue, Zagreb, 10000, Croatia |
| - Central Ethics Committee, Ksaverska cesta 4, Zagreb, 10000, Croatia |
| - Drug Committee Osijek, 4 J.Huttler Street, Osijek, 31000, Croatia |
|  |
| **Czech Republic** |
| - Eticka komise Fakultni nemocnice Ostrava, 17. listopadu 1790, Ostrava 708 52, Czech Republic |
| - Eticka komise Mestske nemocnice Ostrava, Nemocnicni 20, Ostrava 728 80, Czech Republic |
| - Eticka komise Krajske zdravotni, a.s. - Nemocnice Teplice, o.z., Duchcovska 53, Teplice 415 29, Czech Republic |
| - Eticka komise Fakultni nemocnice Olomouc a LF UP v Olomouci, I. P. Pavlova 6, Olomouc 775 20, Czech Republic |
| - Eticka komise Fakultni nemocnice v Motole, V Uvalu 84, Praha 5, 150 06, Czech Republic |
| - Eticka komise Fakultni nemocnice Brno, Jihlavska 20, Brno 625 00, Czech Republic |
| - Eticka komise Vseobecne fakultni nemocnice v Praze, Na Bojisti 1, Praha 2, 128 08, Czech Republic |
|  |
| **Estonia** |
| - Tallinn Medical Research Ethics Committee, Hiiu 42, National Institute for Health Development, Room 124, Tallinn EE-11619, Estonia |
|  |
| **France** |
| - CPP Sud-Méditerranée II - Hôpital Salvator, 249 Boulevard de Sainte Marguerite, Marseille 13009, France |
|  |
| **Georgia** |
| - Independent Ethics Committee at Ltd “S. Khechinashvili University Clinic”, 33, Chavchavadze Ave, Tbilisi 179, Georgia |
| - Independent Ethics Committee at Petre Sarajishvili Institute of Neurology, 13, Tevdore Mgvdeli street, Floor 3, Tbilisi 112, Georgia |
| - Independent Ethics Committee of Ltd “Research Institute at Clinical Medicine”, 13, Tevdore Mgvdeli street, Tbilisi 112, Georgia |
| - Independent Ethics Committee at Ltd “Samkurnalo Combinati”, 16, Kavtradaze street, Tbilisi 186, Georgia |
|  |
| **Germany** |
| - Ethikkommission an der medizinischen Fakultät der Heinrich-Heine-Universität, Moorenstraße 5, Düsseldorf 40225, Germany |
| - Ethik-Kommission der Ärztekammer Westfalen-Lippe und der Medizinischen Fakultät der WWU Munster, Von-Esmarch-Straße 62, Medizinische Fakultät der Westfälischen Wilhelms-Universität Münster, Münster 48149, Germany |
| - Ethik-Kommission der Bayerischen Landesärztekammer, Mühlbaurstraße 16, München 81677, Germany |
| - Ethik-Kommission bei der Ärztekammer Niedersachsen, Berliner Allee 20, Hannover 30175, Germany |
| - Landesamt für Gesundheit und Soziales Berlin, Fehrbelliner Platz 1, Geschäftsstelle der Ethikkommission des Landes Berlin, Berlin 10707, Germany |
| - Ethikkommission des FB Medizin der Philipps-Universität Marburg, Baldingerstraße, Marburg 35032, Germany |
| - Ethik-Kommission an der Medizinischen Fakultät der Universität Leipzig, Härtelstraße 16–18, Institut für Klinische Pharmakologie, Leipzig 40107, Germany |
| - Ethik-Kommission der Ärztekammer Hamburg, Humboldtstraße 67a, Hamburg 22083, Germany |
| - Ethik-Kommission der Medizinischen Fakultät Friedrich-Alexander-Universität Erlangen-Nürnberg, Krankenhausstraße 12, EG, Raum 106, Erlangen 91054, Germany |
| - Ethik-Kommission der Medizinischen Hochschule Hannover, Carl-Neuberg-Straße 1, Hannover 30625, Germany |
| - Ethikkommission der Landesärztekammer Hessen, Im Vogelsgesang 3, Frankfurt 60488, Germany |
| - Ethik-Kommission bei der Landesärztekammer Baden-Württemberg, Jahnstraße 40, Stuttgart 70597, Germany |
| - Ethikkommission der Medizinischen Fakultät der Ruhr-Universität Bochum, Bürkle-de-la-Camp-Platz 1, BG-Kliniken Bergmannsheil, Bochum 44789, Germany |
| - Ethikkommission der Ärztekammer Nordrhein, Tersteegenstraße 9, Düsseldorf 40474, Germany |
| - Ethik-Kommission der Medizinischen Fakultät der Ludwig-Maximilians Universität, München, Pettenkoferstr. 8a, München 81675, Germany |
|  |
| **Greece** |
| - National Ethics Committee for Clinical Trials, Mesogeion 284, Athens 15562, Greece |
|  |
| **India** |
| - Deenath Mangeshkar Hospital and Research Centre, Near Mhatre Bridge, Intensive Care Unit, Maharashtra, Pune 411004, India |
| - Ethics Committee Poona Hospital Research Centre, 27 Sadashiv Peth, Maharashtra, Pune 411030, India |
| - All India Institute of Medical Sciences, Aurobindo Marg, Ansari Nagar, New Delhi 110029, India |
| - Manavata Professional Ethics Committee, Curie manavata cancer centre, Opposite Hotel Sandeep, Near Mahamarg Bus stand, Mumbai Naka, Nashik 422004, India |
| - KMCH Ethics Committee, Avanashi Road, Tamil Nadu, Coimbatore 641014, India |
| - Ethics Committee, Jaslok Hospital and Research Centre, 15, Dr.G.Deshmukh Marg, Maharashtra, Mumbai 40026, India |
| - Ethics Committee, Rajinder Nagar, New Delhi 110060, India |
| - Ethics Committee Vidyasagar Institute of Mental Health and Neuro-Sciences, 1, Institutional Area, New Delhi, Nehru Nagar 110065, India |
| - Institutional Ethics Committee Manipal Hospital and Manipal Heart Foundation, Airport Road, Department of Vascular Surgery, 98, Rustom Bagh Road, Karnataka, Bangalore, 560017, India |
| - Central India Medical Research Ethics Committee, Dr.S.M.Patil's Hospital, 2nd Floor, Yugadharma Complex, Ramdaspeth, Nagpur 440010, India |
| - Independent Ethics Committee, TN Medical College and BYL Nair Ch. Hospital, Dept of Clinical Pharmacology, Old RMO Bldg, Mumbai 400008, India |
| - Max Healthcare Ethics Committee, 1, Press Enclave Raod, Saket 110017, India |
| - Independent Ethics Committee, Cerebrovascular and Vasculities Research Foundation, Flat 'B' Balaji Villa, 9/2, Rajarathinam Street, Kalipauk 600 010, India |
| - G.K. Hospital Ethics Committee for Human Subject Research, 11/2 Old Palasia, Department of Neurology, Indore 452018, India |
| - Central Ethical Committee, Medical Sciences Complex, Nitte University, Mangalore 575018, India |
| - Ethics committee - SMS Medical College and attached Hospital, Jawaharlal Nehru Marg, Jaipur 302004, India |
| - Well Care Research Ethics Committee, 25 New Jagnath Road, Behind A.G Office, Gujarat, Rajkot 360001, India |
| - Sujlam Independent Ethics Committee, 2nd, floor, AMA House, Near Natraj Cinema, Ashram Road, Ahmedabad 380009, India |
| - The Ethics Committee Of Sri Aurobindo Seva Kendra, Sri Aurobindo Seva Kendra, 1H, Gariahat road (South), Kolkata 700068, India |
| - SAHEB Central Ethics Committee, Amritsar, 1st floor, 143-144/7, Near Gurunanak Bhawan, City Centre Market, Amritsar 143001, India |
| - Bangalore Central Ethics Committee, No. 1423, Kullappa Circle, Kullappa Layout, St. Thomas Town, Kammanahalli, Bangalore 560084, India |
|  |
| **Latvia** |
| - The Ethics Committee for Clinical Trials on Medicinal Products, Aizkraukles Street 21-113, Riga LV-1006, Latvia |
|  |
| **Mexico** |
| - Comite de Etica e Investigacion del "Instituto Biomedico de Investigacion A.C.", Sierra Fria 218, Fraccionamiento Bosques del Prado Norte, Aguascalientes 20217, Mexico |
| - Comite de Bioetica del Instituto de Ciencias Biomedicas Angeles, Camino a Santa Teresa 1055, Torre Especialidades, Colonia Heroes de Padierna, Mexico City, DF 10700, Mexico |
| - Comite Bioetico para la Investigacion Clinica S.C. Institutional Review Board, Puebla 422 despacho 4, Col. Roma Sur, Mexico, DF 6700, Mexico |
| - Comision de Ética del Hospital San José Tec de Monterrey y de la División de Ciencias de la Salud, Av. I. Morones Prieto 3000 Pte, Despacho 1, Col. Los Doctores, Monterrey, Nuevo Leon 64170, Mexico |
| - Tijuana General Hospital, Comite de Ensenanza e Investigacion, Aveinda Centenario Numero 10851 Zona Rio, Instituto de Servicios de Salud Publica Del Estado de Baja California, Tijuana, Baja California 22320, Mexico |
| - Comité de Ética e Investigación Christus Muguerza del Parque SA de CV, Calle Dr. Pedro Leal Rodriguez 1802, Colonia Centro, Chihuahua 31000, Mexico |
|  |
| **The** **Netherlands** |
| - AZM METC, Oxfordlaan 10, Kamer 4.R1.33, Maastricht 6202 AZ, Netherlands |
|  |
| **New Zealand** |
| - Multi-Region Ethics Committee, PO Box 5013, Level 2, 1-3 The Terrace, Wellington, New Zealand |
|  |
| **Peru** |
| - Comite de Etica para la Investigacion de la Universidad de San Martin de Porres Clinica CADAMUJER, Avenida Alameda del Corregidor 1531, Urbanizacion Los Sirius, Las Viñas, La Molina, Lima 12, Peru |
| - Comité de Ética en Investigación Biomédica del Hospital Nacional “Dos de Mayo”, Parque Historia de La Medicina Peruana, s/n, Av. Grau, Cuadra 13, Lima, Lima 01, Peru |
|  |
| **Poland** |
| - Komisja Bioetyki Uniwersytetu Medycznego w Lodzi, Kosciuszki 4, Lodz 90-419, Poland |
|  |
| **Romania** |
| - National Ethics Committee for Clinical Trial on Medicine, 48 Aviator Sanatescu Street, Sector 1, Bucharest 11478, Romania |
|  |
| **Russia** |
| - Ethics Committee at the Federal Service on Surveillance in Healthcare and Social Development of RF, Petrovskiy Bulvar, 8, stroenie 2, Moscow 127051, Russia |
| - Ethics Committee at Siberian Regional Medical Centre, Ulitsa Kainskaya, 13, Novosibirsk 630007, Russia |
| - Ethics Committee within Chelyabinsk City Clinical Hospital #3, Prospect Pobedy, 287, Chelyabinsk 454136, Russia |
| - Ethics Committee at Republican Clinical Hospital for Rehabilitation Treatment, Ulitsa Vatutina, 13, Kazan 420021, Russia |
| - Ethics Committee within Bashkiria State Medical University, Ulitsa Lenina, 3, Ufa 450000, Russia |
| - Ethics Committee within Smolensk Regional Clinical Hospital, Prospect Gagarina, 27, Smolensk 214018, Russia |
| - Ethics Committee at City Clinical Hospital # 11, Ulitsa Dvintsev, 6, Moscow 127018, Russia |
| - Ethics Committee within Central Clinical Hospital #2 n.a. N.A. Semashko OAO "RZhD", Ulitsa Budayskaya, 2, Moscow 129128, Russia |
| - Ethics Committee within Siberian State Medical University, Moscovskiy Tract, 2, Tomsk 634050, Russia |
| - Ethics Committee within Moscow Medical Academy n.a. I.M. Sechenov, 8 Ulitsa Trubetskaya stroenie 2, City Clinical Hospital #61, Moscow 119992, Russia |
| - Ethics Committee at City Hospital #2 – Kransodar Multispeciality Treatment and Diagnostics Unit, Ulitsa Krasnykh Partizan, 6, korp. 2, Krasnodar 350012, Russia |
| - Ethics Committee at Perm State Medical Academy, Ulitsa Kuybysheva, 39, Department of General Surgery, Perm 614990, Russia |
| - Ethics Committee within Research Institute of Neurology of RAMS, Volokolamskoye Shosse, 80, Moscow 125367, Russia |
|  |
| **Serbia** |
| - Local Ethics Comittee at Clinical Center NIS, 48 Zorana Djindjica Boulevard, Nis 18000, Serbia |
| - Local Ethics Committee at Military Medical Academy, 17 Crnotravska Street, Belgrade 11000, Serbia |
| - Local Ethics Comittee at Clinical Center of Serbia, 2 Pasterova Street, Belgrade 11000, Serbia |
| - Local Ethics Comittee at Clinical Hospital Center "Kragujevac", 30 Zmaj Jovina Street, Kragujevac 34000, Serbia |
|  |
| **Spain** |
| - CEIC Hospital La Paz, Paseo de la Castellana, 261, Hospital General - Comité Ético de Investigación Clínica, Planta 8, Madrid 28046, Spain |
| - CEIC de Andalucía, Avenida de la Innovación s/n, Servicio de Investigación y Desarrollo Personal, Edificio Arena 1, Consejería de Salud - Dirección General de Procesos y Formación, Sevilla 41020, Spain |
| - CEIC Hospital Virgen Macarena, Calle Dr. Fedriani, 3, Comité de Ensayos Clínicos, Planta 2, Sevilla 41009, Spain |
| - CEIC Hospital Universitario Reina Sofía, Avenida Menéndez Pidal, s/n, Planta 1, Edificio de Consultas Externas, Córdoba 14004, Spain |
| - Instituto de Investigación Hospital 12 de Octubre (i+12), Avenida de Córdoba s/n, Area de Gestión de Proyectos - Unidad Administrativa CEIC, Planta 6ª, Centro de Actividades Ambulatorias - Bloque D, Madrid 28041, Spain |
|  |
| **Ukraine** |
| - Central Commission on Ethics Questions of the MoH of Ukraine, Vulytsya Narodnogo Opolchennya, 5, Kyiv 3680, Ukraine |
| - Committee on Ethic Questions of Kyiv City Clinical Hospital #4, Vulytsya Solomyanska, 17, Kyiv 3110, Ukraine |
| - Commission on Ethics Questions of Municipal Institution of Healthcare Kyiv Regional Clinical Hospital, Vulytsya Baggovutivska, 1, Thoraco-Pulmonary Centre, Kyiv 4107, Ukraine |
| - Commission on Biomedical Ethics Questions of Chernivtsi Regional Psychiatric Hospital, Vulytsya Musorgskogo, 2, Chernivtsi 58018, Ukraine |
| - Commission on Ethics Questions of Crimean Republican Institution Clinical Hospital n.a N.A. Semashko, Vulytsya Kyivska, 69, Simferopol 96017, Ukraine |
| - Bioethics Committee within Dnipropetrovsk State Medical Academy, Dzerzhinskogo vulytsya, 9, Dnipropetrovsk 49044, Ukraine |
| - Ethics Commission of Ukrainian State Research Institute of Medical and Social Problems of Disability, Provulok Radyanskyy, 1a, Dnipropetrovsk 49027, Ukraine |
| - Committee on Medical Ethics of Central Clinical Hospital of Railways of Ukraine, Provulok Balakireva, 5, Kharkiv 61103, Ukraine |
| - Commission on Bioethic Questions of Donetsk National Medical University named after M. Horkyy, Prospekt Illicha, 16, Donetsk 83003, Ukraine |
| - Comission on Ethics Questions of Vinnytsa Regional Psychoneurological Hospital n.a. Yuschenko, Vulytsya Pyrogova, 109, Vinnytsya 21005, Ukraine |
| - Commission on Ethics Questions of Odesa Regional Clinical Hospital, Vulytsya Zabolotnogo, 26, Odesa 65117, Ukraine |
| - Commission on Ethics Questions of Poltava Regional Clinical Hospital n.a. M.V. Sklifosovskyy, Vulytsya Shevchenka, 23, Poltava 36024, Ukraine |
| - Local Ethic Commission of Institute of Neurology, Psychiatry and Narcology AMS of Ukraine, Vulytsya Akademika Pavlova, 46, Kharkiv 61068, Ukraine |
| - Commission on Ethics Questions of Ternopil Regional Municipal Clinical Psychoneurological Hospital, Vulytsya Troleybusna, 14, Ternopil 46027, Ukraine |
|  |
| **United Kingdom** |
| - West of Scotland Research Ethics Committee 1, Dumbarton Road, Western Infirmary, Glasgow, G11 6NT, United Kingdom |
|  |
| **United States** |
| - Chesapeake Research Review Incorporated Institutional Review Board, 7063 Columbia Gateway Drive, Suite 110, Columbia, MD 21046, United States |
| - Johns Hopkins Medicine Institutional Review Board, 1620 McElderry Street, Reed Hall, Suite B-130, Baltimore, MD 21205-1911, United States |
| - Western IRB, 3535 Seventh Avenue Southwest, Olympia, WA 98502-5010, United States |
| - Saint Joseph's Hospital and Medical Center Institutional Review Board, 350 West Thomas Road, Phoenix, AZ 85013, United States |
| - Mercy Medical Center Institutional Review Committee, 1111 Sixth Avenue, Des Moines, IA 50314, United States |
| - Cleveland Clinic Foundation Regulatory Committee, 9500 Euclid Avenue, Desk HSb 103, Cleveland, OH 44195, United States |
