# Supplementary material for: Effect of peginterferon beta-1a on MRI measures and achieving no evidence of disease activity: results from a randomized controlled trial in relapsing-remitting multiple sclerosis
Source: BMC Neurol. 2014 Dec 31;14:240. doi: 10.1186/s12883-014-0240-x (PMC4311432; doi:10.1186/s12883-014-0240-x)
Supplement: Additional file 2: Figure S1. — Lesion numbers at Weeks 24 and 48. Figure S2. MRI-NEDA proportions. Figure S3. Clinical-NEDA proportions. [file 12883_2014_240_MOESM2_ESM.docx]

**Additional File 2
Figures


Additional File 2 Figure 1.** Lesion numbers at Weeks 24 and 48

New active lesions (sum of Gd+ plus non-enhancing new or newly-enlarging T2 hyperintense) compared to baseline


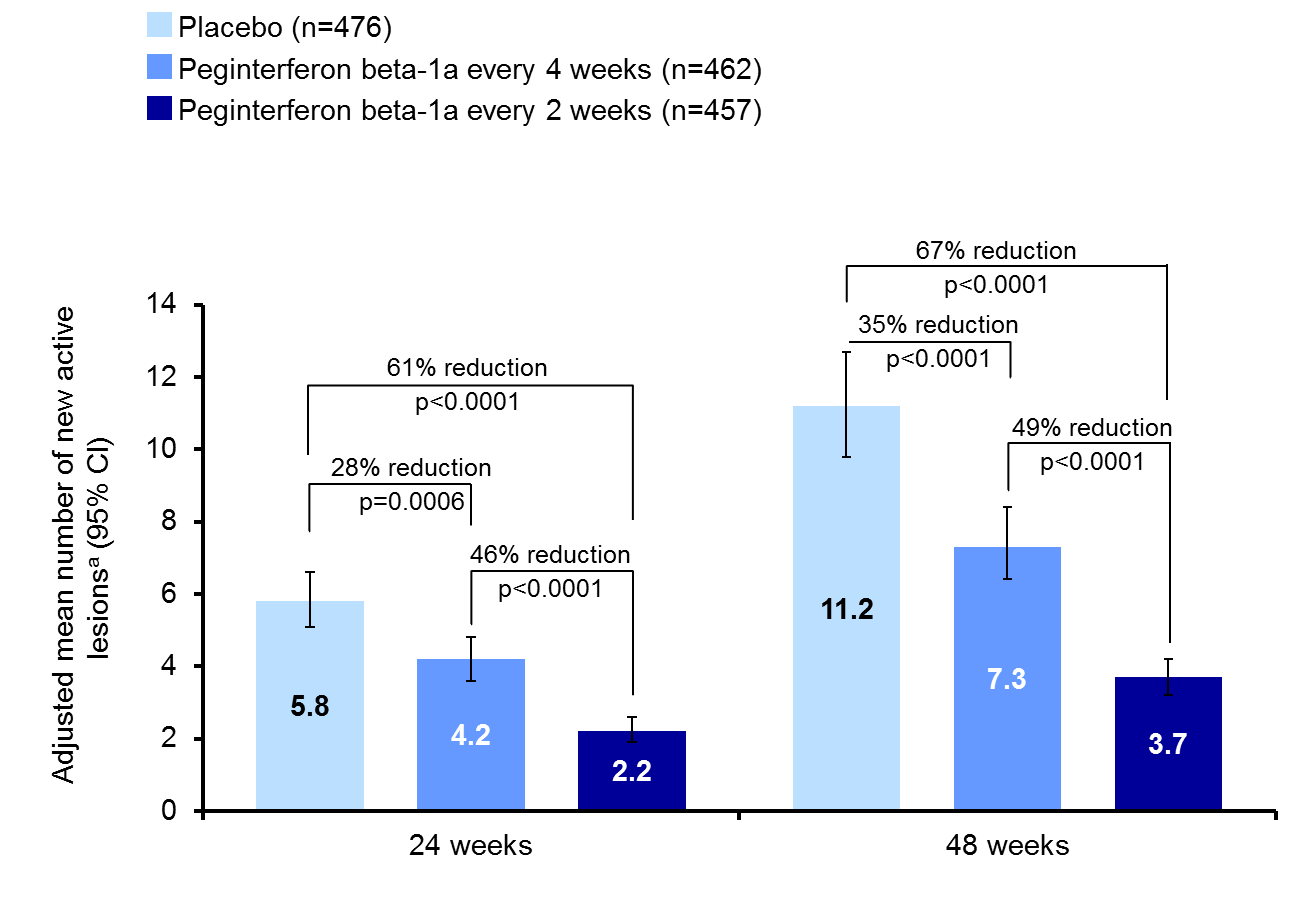


^a^Based on mean number of new lesions. Negative binomial regression analysis, adjusted for baseline number of Gd+ lesions. The data for 48 weeks has been published previously [10]. CI = confidence interval.

**Additional File 2 Figure 2.** MRI-NEDA proportions

1. Baseline to Week 48


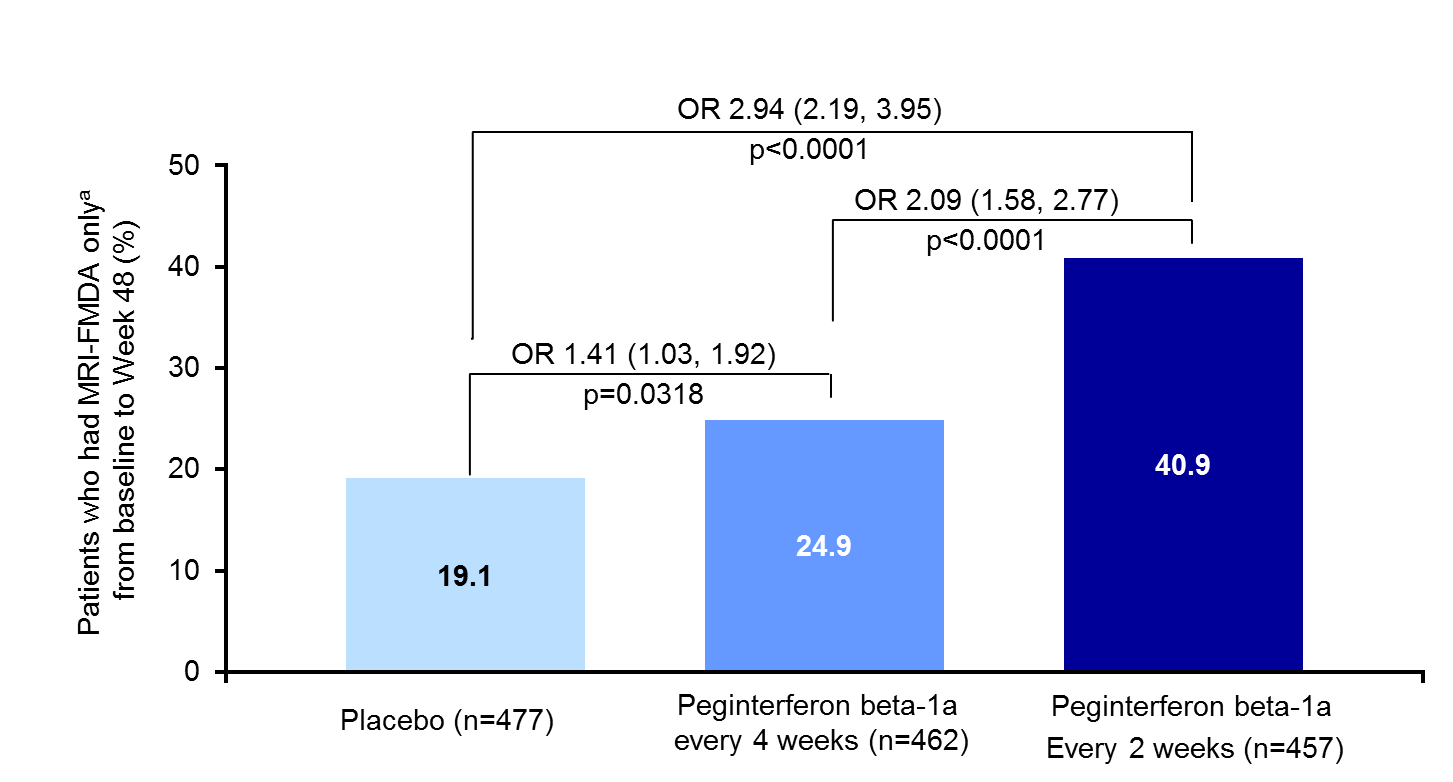


1. Baseline to Week 24


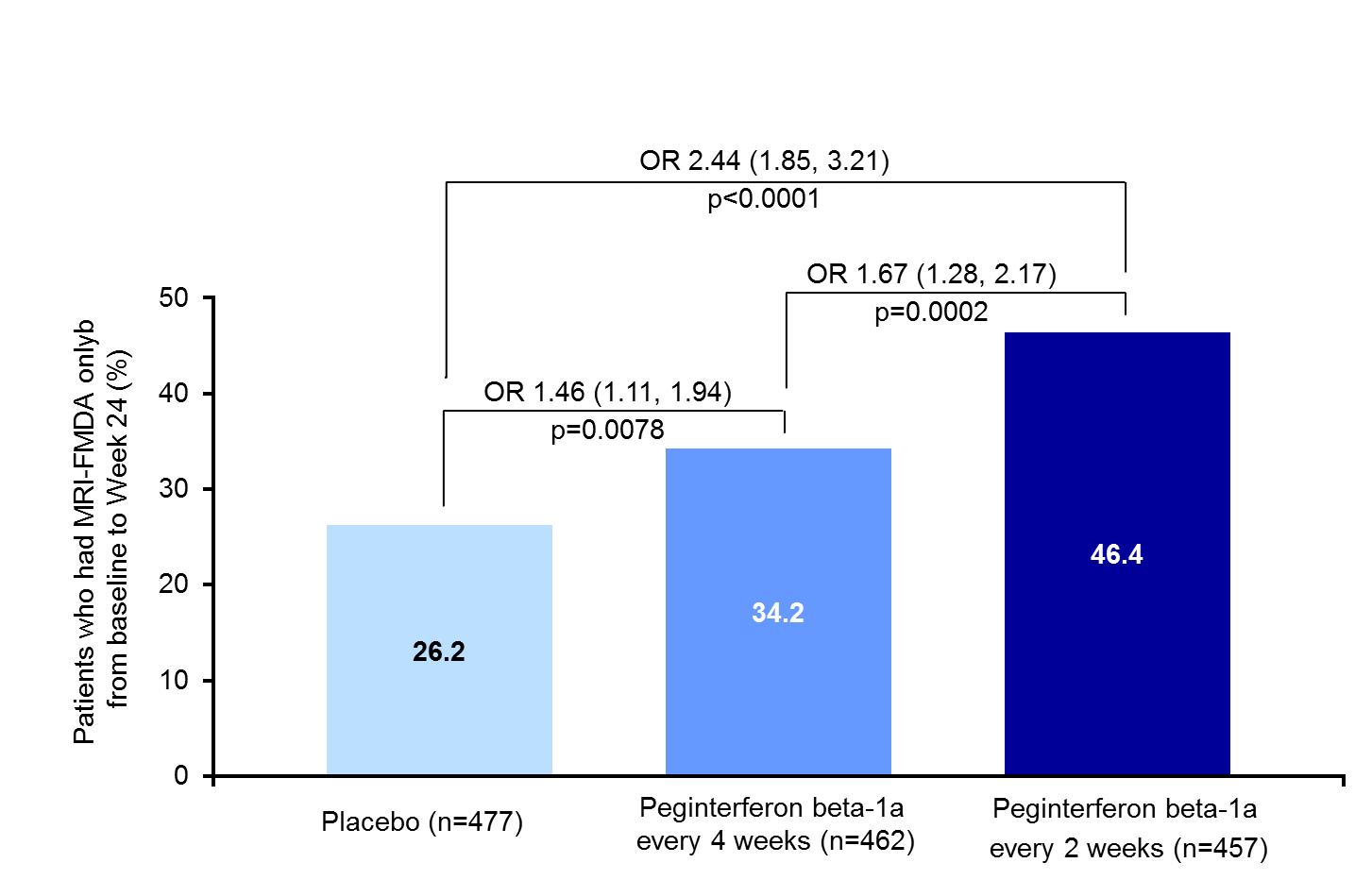


1. Weeks 24−48


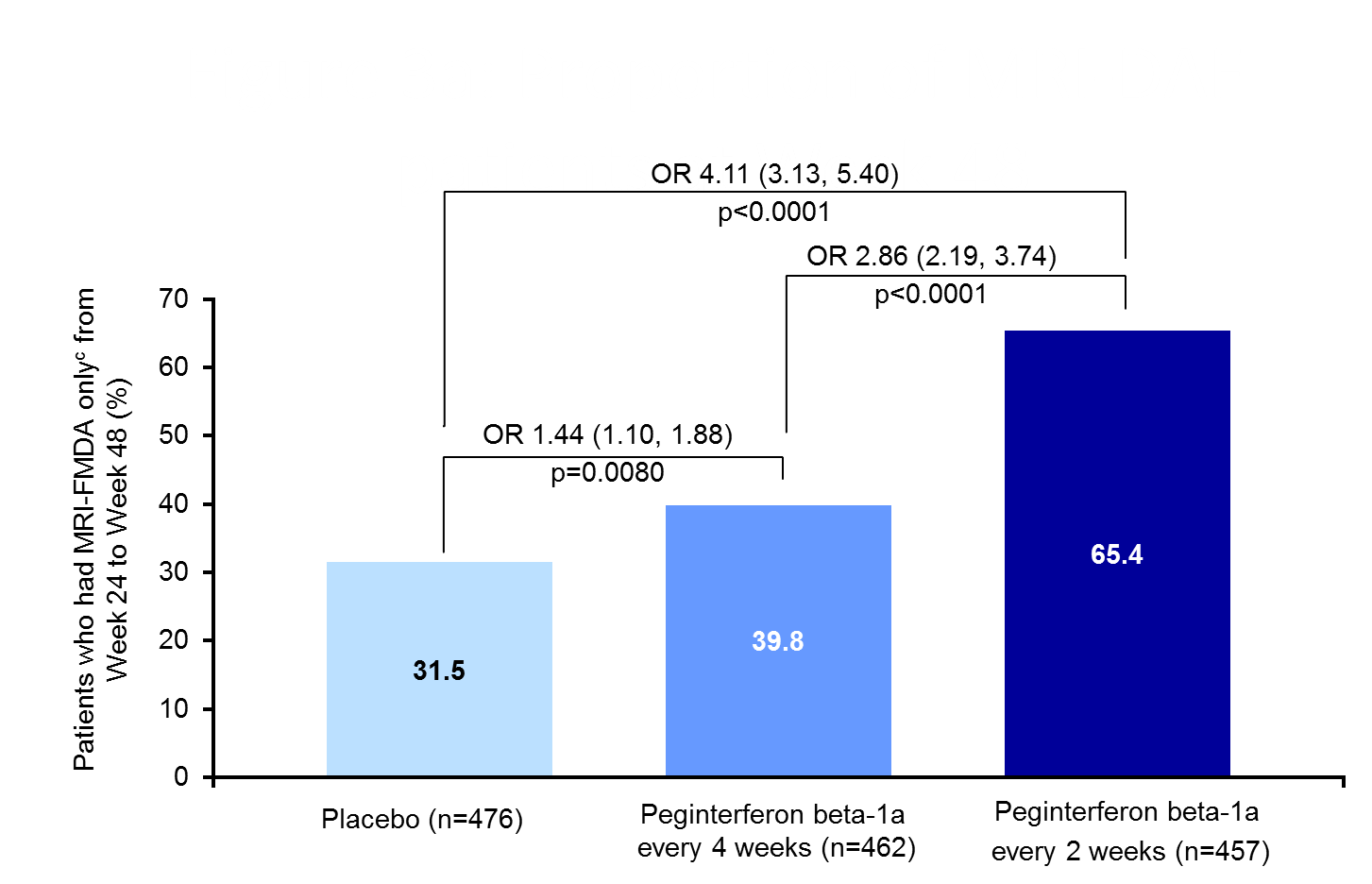


ORs are shown with 95% confidence intervals in parentheses. ^a^Defined as no gadolinium-enhancing lesions at Week 24 and Week 48 and no new or newly-enlarging T2 lesions compared with baseline at Week 48. ^b^Defined as no gadolinium-enhancing lesions at Week 24 and no new or newly-enlarging T2 lesions at Week 24 compared with baseline; ^c^Defined as no gadolinium-enhancing lesions at Week 48 and no new or newly-enlarging T2 lesions at Week 48 compared with Week 24 or the closest previous visit before Week 48. Data from patients with complete MRI results during the time interval were used. NEDA=No evidence of disease activity; MRI = magnetic resonance imaging; OR = odds ratio.

**Additional File 2 Figure 3.** Clinical-NEDA proportions

1. Baseline to Week 48


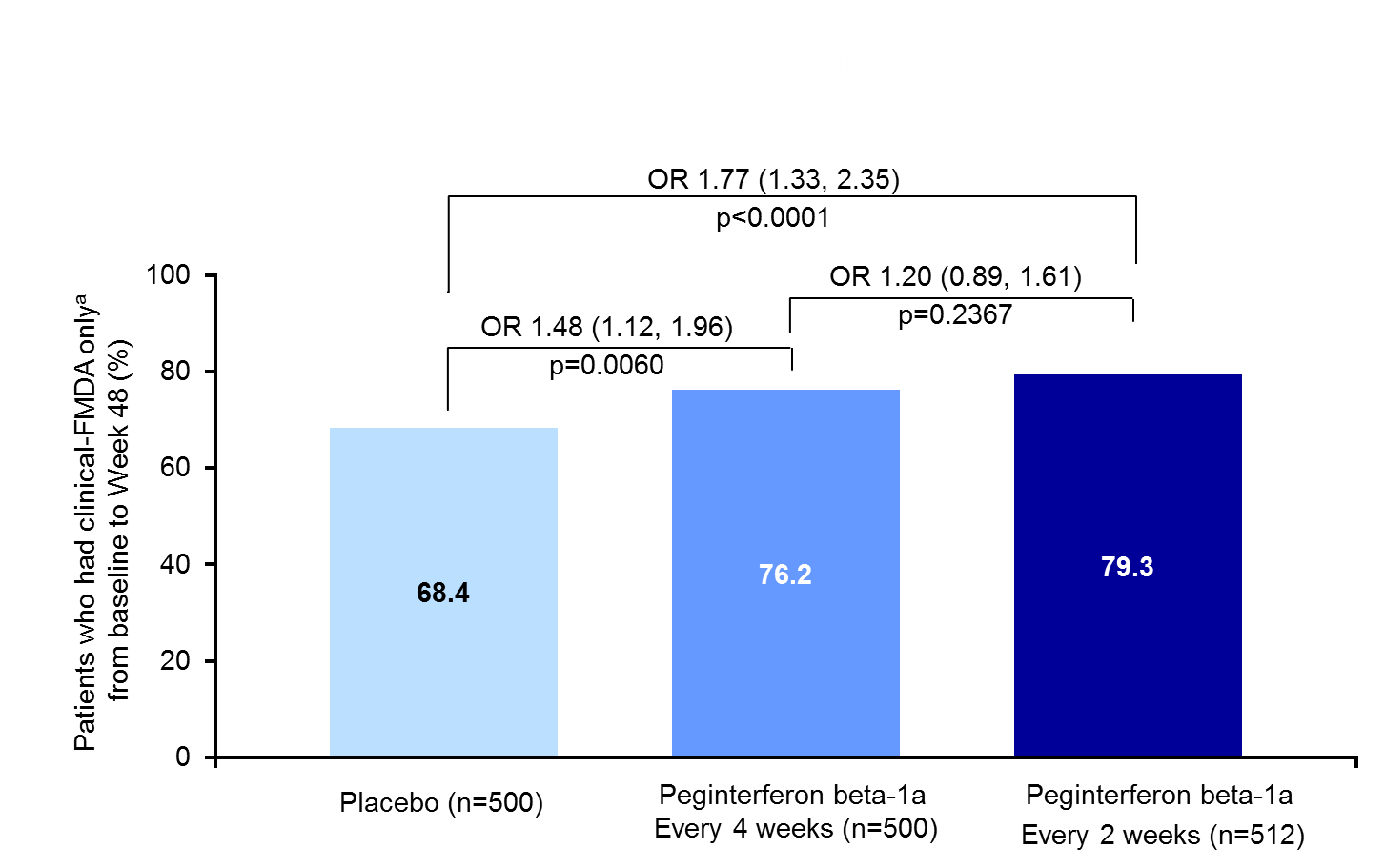


1. Baseline to Week 24


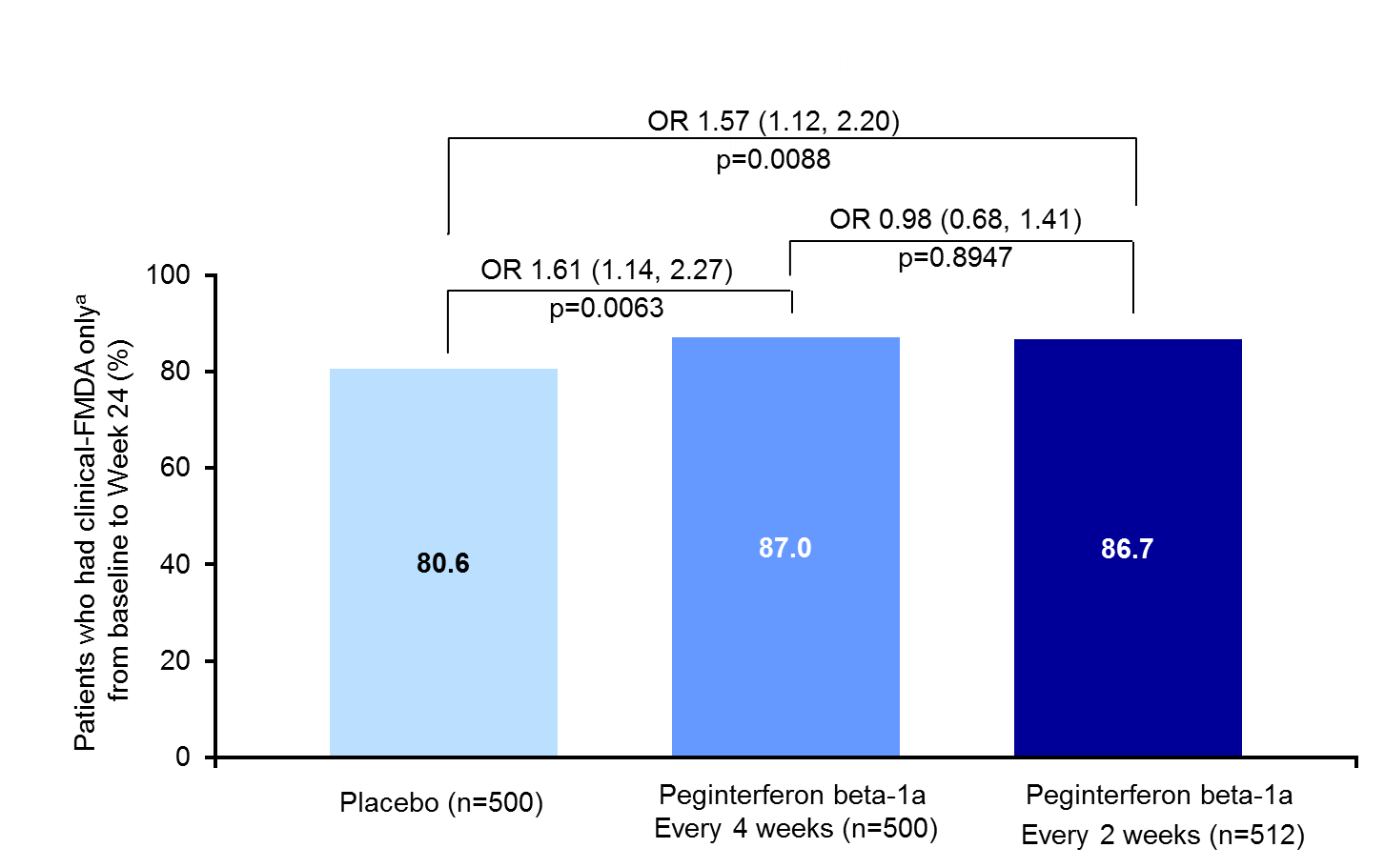


1. Weeks 24−48


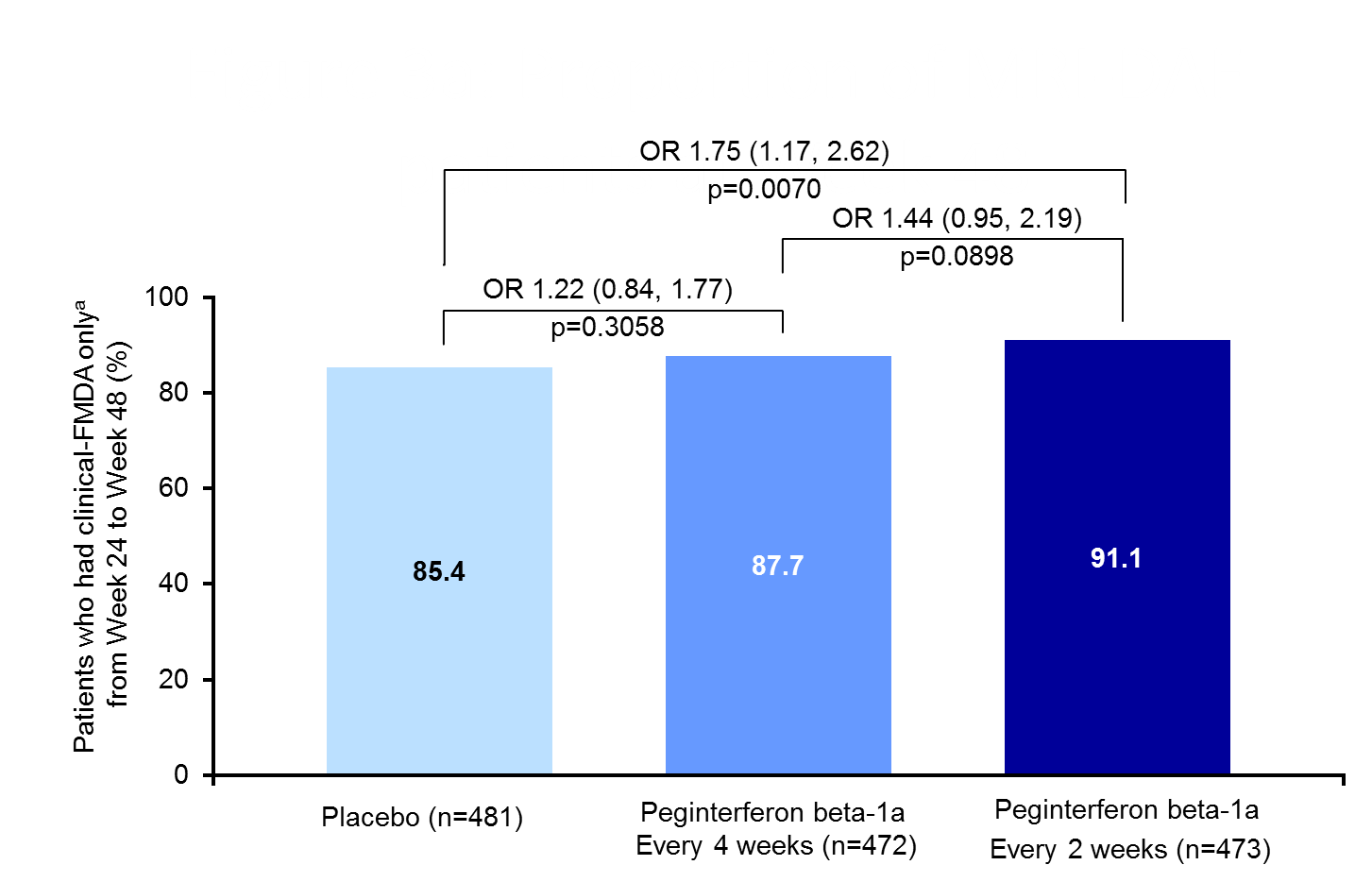


ORs are shown with 95% confidence intervals in parentheses. **^a^**Defined as no relapses and no onset of 12-week confirmed disability progression over the interval specified. NEDA = No evidence of disease activity; OR = odds ratio.
